# Applications of CRISPR-Cas-Based Genome Editing Approaches Against Human Cytomegalovirus Infection

**DOI:** 10.3390/biomedicines13071590

**Published:** 2025-06-30

**Authors:** Andra Zhang, Isadora Zhang, Fenyong Liu

**Affiliations:** 1 School of Public Health, University of California, Berkeley, CA 94720, USA; 2 Department of Molecular Cell Biology, University of California, Berkeley, CA 94720, USA; 3 Program in Comparative Biochemistry, University of California, Berkeley, CA 94720, USA

**Keywords:** clustered regularly interspaced short palindromic repeats (CRISPR), CRISPR-associated protein (Cas), cytomegalovirus, herpesvirus, genome-editing, gene-editing, gene-targeting, gene therapy, antiviral

## Abstract

Human cytomegalovirus (HCMV), a globally ubiquitous herpesvirus with the ability to carry out both lytic productive and lifelong latent infections, is a major cause of congenital infections, often leading to intellectual disabilities and neurological disorders. Moreover, HCMV is an opportunistic pathogen commonly found in immunocompromised individuals such as organ transplant recipients, HIV-positive individuals, and cancer patients, causing severe and life-threatening complications. While effective in inhibiting viral lytic infection, current FDA-approved compounds cannot eliminate the latent viral genome and have little effect on viral latent infection. Developing novel antiviral therapeutic approaches to eliminate HCMV lytic and latent infections is a major public health priority for controlling HCMV infection and preventing viral-associated diseases. The genome-editing technology based on the Clustered Regularly Interspaced Short Palindromic Repeats (CRISPR)/CRISPR-associated protein (Cas) RNA-guided nuclease system represents a novel and promising antiviral approach through modifying or destroying the genetic material of human viruses. This review summarizes the recently published progress in using the CRISPR-Cas approach to study and inhibit HCMV infections and discusses prospects for developing the CRISPR-based genome-editing technology for therapeutic applications against HCMV infection and associated diseases.

## 1. Cytomegalovirus

Cytomegalovirus (CMV) is a member of the herpesvirus family, specifically belonging to the β-herpesvirus subfamily [1]. Among the various species of CMV found in different animals, human cytomegalovirus (HCMV), also known as human herpesvirus 5 (HHV-5), is the most studied [2,3]. HCMV can be transmitted from mother to child congenitally, during birth, or via breastfeeding [4]. Congenital infection of HCMV is a major cause of neonatal morbidity and can cause several complications including pneumonia, gastrointestinal liver damage, and central nervous system damage [5,6]. In severe cases, it can even lead to neonatal death [4]. Additionally, HCMV can also be transmitted through blood transfusions, organ transplantation, close contact with infected hosts, and sexual activity [7]. Immunocompromised individuals, such as transplant recipients and patients with acquired immunodeficiency syndrome (AIDS), are especially vulnerable as their weakened immune systems are less capable of containing the virus, resulting in significant clinical concerns [3].

The HCMV genome is more than 230 kb long, making it the largest of all human herpesviruses [1,8,9]. HCMV’s large genome size gives it the ability to encode at least 200 genes, including many immune evasion genes, which function to interfere with the innate and adaptive immune systems, immune effector functions, and other signaling pathways [3,9]. In addition to encoding immune evasion genes, HCMV, like all other herpesviruses, has the ability to establish lifelong infections in the host. The virus can enter the latent phase, where it remains dormant and is not actively producing virions [3]. Upon infecting a host, HCMV can enter polymorphonuclear leukocytes (PMNs) or monocytes in the blood and establish infection. These infected cells carry the virus along the bloodstream, allowing the virus to spread to various organs in the body. Once the virus reaches the bone marrow, it infects hematopoietic progenitor cells and specifically exploits monocytes and other cells of the myeloid lineage to establish latency [9]. In the latent stage, HCMV effectively evades the immune system and is asymptomatic in the infected host [3]. HCMV typically maintains its latent phase within healthy individuals; however, for immunocompromised individuals, such as AIDS patients and transplant recipients, the virus can reactivate and enter the lytic phase, where it takes over host cells to actively replicate and cause diseases [10].

Antiviral drugs used to treat HCMV include Ganciclovir or its oral prodrugs Valganciclovir, Foscarnet, and Cidofovir, which are herpesvirus DNA polymerase inhibitors that suppress HCMV replication [11,12,13]. However, their clinical usage is often limited by their significant toxicity in the human body [3]. Ganciclovir and Valganciclovir are associated with neutropenia, a condition characterized by a decreased number of neutrophils, which increases the risk of secondary infections [14]. Foscarnet and Cidofovir are associated with nephrotoxicity, which can cause long-term kidney injury [15]. Furthermore, these FDA-approved drugs have little effect on HCMV latent infection and cannot completely eliminate latent viral DNA genomes. These inadequacies of existing treatments for HCMV highlight the need for safer and more effective therapeutic strategies against this virus.

## 2. Clustered Regularly Interspaced Short Palindromic Repeats (CRISPR)/CRISPR-Associated Protein (Cas) Gene-Editing System

Over the past decades, genome-editing technologies have rapidly advanced, with the CRISPR-Cas gene-editing system emerging as a transformative and widely adopted tool due to its simplicity and versatility [16,17,18]. CRISPR (Clustered Regularly Interspaced Short Palindromic Repeats), along with CRISPR-associated (Cas) genes, is a naturally occurring adaptive immune system found in bacteria and archaea that protects them from viral infections [19]. The CRISPR locus consists of short, repeating DNA sequences separated by unique spacer sequences. The repeats prevent bacteria and archaea from cleaving self-DNA and help with integrating new spacers while the spacer sequences, fragments of viral or plasmid DNA captured from invaders, serve as memory of past infections [20].

**Figure 1:**
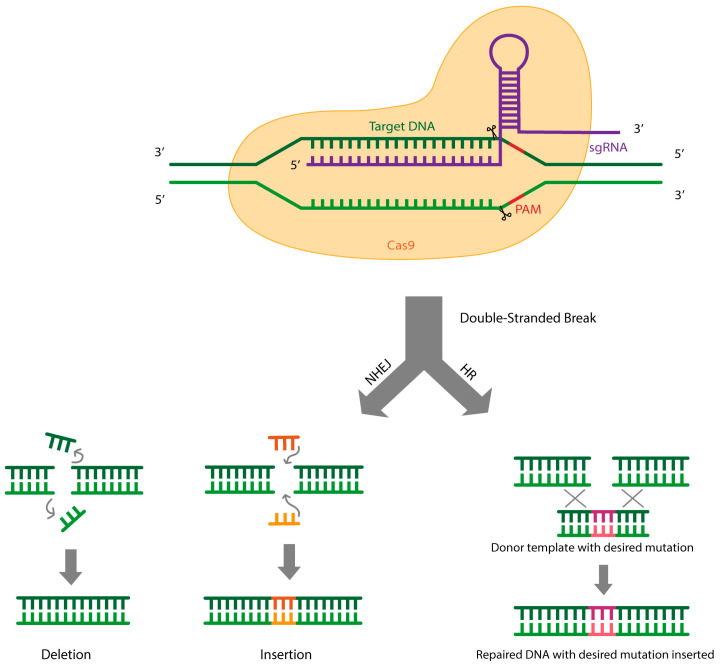
CRISPR-Cas9 gene-editing system. The Cas9 endonuclease is guided by a sgRNA to the location of the target genomic sequence, where the 5′ end of the sgRNA binds to the target sequence. Upon recognition of the PAM site located downstream of the target sequence, Cas9 cleaves target DNA and introduces a double-strand break. The cleaved DNA can then be repaired via non-homologous end-joining (NHEJ) or homologous recombination (HR) processes.

Many bacteria use the type II CRISPR-Cas system, which utilizes the Cas9 endonuclease [16,17,18]. Cas9 acts together with a guide RNA to recognize and cleave foreign DNA by recognizing short, conserved sequences called PAMs (Proto-spacer Adjacent Motifs), which are found in foreign DNA (Figure 1). For effective targeting of the CRISPR-Cas9 method, Cas9 requires the RNA duplex formed by crRNA (CRISPR RNA), which guides Cas9 to the correct target DNA, and tracrRNA (trans-acting CRISPR RNA), which plays a role in crRNA maturation and Cas9 expression [21]. Researchers have been able to fuse the crRNA and tracrRNA into a single guide RNA (sgRNA), which contains a 20-nucleotide sequence at the 5′ end that identifies the DNA target site via Watson–Crick base pairing and a 3′ duplex RNA structure that facilitates the binding of Cas9 (Figure 1) [22]. This innovation greatly enhanced the efficiency and simplicity of the CRISPR-Cas9 gene-editing system, enabling it to become a powerful tool in genome-editing technology in both research and therapeutic applications [16,17,18].

When Cas9 introduces a site-specific double-strand break (DSB) in the target DNA, the damaged DNA can be repaired in two ways (Figure 1). The first is through homologous recombination (HR), where genetic information is exchanged between two identical chromatids [16,17,18]. HR is commonly used to introduce specific genetic changes, such as inserting new sequences or replacing existing ones by providing a repair template that contains the desired modification sequence flanked by regions of homology to the target site (Figure 1). In contrast, in the second repair pathway, non-homologous end-joining (NHEJ), broken DNA ends are directly ligated together without the need for a template [16,17,18]. The NHEJ process is considered the more error-prone pathway, as it tends to introduce random insertions or deletions (indels) at the repair site (Figure 1) [22]. Researchers take advantage of both repair mechanisms to modify the target genome, enabling a wide range of applications in both research settings and clinical applications. For example, using the CRISPR-Cas9 system to induce precise DSBs allows for the creation of human cell lines that resemble various types of cancers, aiding the process of researching the development or progression of those cancers. The CRISPR-Cas9 system can also serve as a screening method in genome-wide studies, such as by knocking out different genes in cancer cells to identify the effects of those genes on cancer cell survival [22].

The versatility of CRISPR-Cas has opened new possibilities for studying and treating infectious diseases, including HCMV. Given the limitations associated with current anti-HCMV drugs, it is important to identify more suitable ways to combat HCMV. In this review, we will summarize the recent progress on using the CRISPR-Cas methods as both a research tool and a therapeutic strategy for HCMV infections.

## 3. Developing CRISPR/Cas-Based Technologies for the Studies of HCMV Infection

One useful way to understand HCMV biology is performing reverse genetic analysis, which requires researchers to genetically engineer the virus. Traditionally, this process uses bacterial artificial chromosome (BAC)-mediated recombination techniques [23,24]. However, this process has its own limitations, such as the lengthy time required to complete the process due to the large genome size of HCMV and the long replication cycle of HCMV [25]. To overcome these challenges, King and Munger reported the use of the CRISPR/Cas9 method to deliver precise modifications onto the HCMV genome and evaluated the effects resulting from both the homologous recombination (HR) and NHEJ processes [26].

To evaluate the possibility of using the CRISPR-Cas9 approach to introduce large insertions into the genome, King and Munger created a construct with a GFP–blasticidin deaminase fusion gene (GFP-BSDR) containing HCMV-specific homologous sequences on both ends to promote the incorporation of new sequences via the HR process [26]. The DNAs encoding Cas9 and a gRNA targeting a non-essential region between US34 and TRS1 were transduced into human MRC5 cells using a lentiviral vector (Table 1). These cells were infected with HCMV and transfected with the GFP-BSDR template, resulting in successful insertion of the large sequence into the viral genome [26]. To evaluate the relative effects of the NHEJ and HR processes, King and Munger focused on DNA Ligase IV, a key enzyme involved in the NHEJ process [26]. By using the DNA Ligase IV inhibitor, SCR7, they aimed to suppress the NHEJ process and promote HR-mediated gene knockout mutations. While successful GFP knockout mutations were observed, they found that they were not a result of the HR process. Rather, the gene knockout efficiency of over 75% was accomplished with NHEJ-mediated mutations. After optimizing various parameters to test HR efficiency, they found that the HR process could achieve over 40% recombination efficiency [26]. These results show that CRISPR-Cas9-mediated methods can be a promising technique for CMV genome mutagenesis, and thus a useful tool in studying CMV gene functions.

In a different study, Kowalik and colleagues performed genome-wide CRISPR-Cas9 screens to identify host genes essential for HCMV infection [27]. The researchers used cells that consistently express Cas9 and a sgRNA library, which targeted approximately 19,050 human genes, with each gene being targeted by 6 sgRNAs. Human epithelial cells and fibroblast cells were repeatedly exposed to HCMV TB40E strain and AD169 strain, respectively, over a three-month period. Cell survival of infection was used as an indicator of potential inactivation of a host gene essential for HCMV infection, preventing HCMV from infecting the cells. This CRISPR-Cas screen identified ORF14I1, a gene encoding an olfactory receptor family membrane protein, as an essential gene for epithelial cell infection by HCMV [27]. These findings suggested a novel host factor that could serve as a therapeutic target for HCMV infection. The use of a CRISPR-Cas screen enabled identification and investigation of host genes that contribute to HCMV infection while offering more precision and flexibility compared to other gene-targeting approaches such as RNA interference (RNAi), which may result in incomplete knockouts [27,28]. This study exemplifies how the CRISPR-Cas9 system can accelerate the discovery of novel essential genes that can be used to develop HCMV therapies.

**Table 1:** HCMV genes targeted by the CRISPR-Cas method and the effects on HCMV replication.

Delivery Vector	Infection Model	Target Genes	Outcome	Reference
Lentivirus	In Vitro (MRC5 cells)	Essential genes: UL54, UL44, UL57, UL70, UL105, UL86, UL84	Impaired viral replication	[29]
		Non-essential genes: US6, US7, and US11	No interference with HCMV replication	
Lentivirus	In Vitro (MRC5 cells)	UL122/123	Decreased IE genes and viral replication	[30]
Lentivirus	In Vitro (MRC5 cells)	Non-essential genes: US7 and US34	No interference with HCMV replication	[26]
Lentivirus	In Vitro (HFF and THP-1 cells)	UL122/123	Significant decreased HCMV replication and reactivation	[31]
Lentivirus	In Vitro (HFF cells)	UL23	Conversion of wild-type viruses into gene drive viruses with attenuation	[32]
Lentivirus	In Vitro (HFF cells)	Essential genes: UL79, UL99	Gene drive viruses had very little spread when mixed with wild-type virus	[33]
		Not essential, but needed for efficient replication: UL26, UL35	UL26: resulted in very poor viral replication	
			UL35: resulted in moderately poor viral replication	

HFF, human foreskin fibroblast. IE, immediate-early.

## 4. Developing CRISPR/Cas-Based Technologies for the Treatment of HCMV Infection

### 4.1. Targeting HCMV Genes Using CRISPR-Cas Approach with Single sgRNAs

One study aimed to demonstrate the feasibility of using CRISPR-Cas9 to target essential viral genes and effectively inhibit HCMV replication. In their study, van Diemen and colleagues designed multiple guide RNAs (gRNAs) to target seven essential HCMV genes: UL54 (viral polymerase), UL44 (polymerase accessory protein), UL57 (single-strand DNA-binding protein), UL70 (primase), UL105 (DNA helicase), UL86 (major capsid protein), and UL84 (initiator protein for viral DNA replication) (Table 1) [29]. They used lentiviral delivery to introduce the gRNAs into MRC5 cells, which were subsequently infected with HCMV that contained an EGFP gene. Through assessing EGFP expression, they found that most gRNAs were capable of impairing viral replication. These results show the potential of using CRISPR-Cas9 as an antiviral strategy, provided that essential genes are targeted [29].

Nonetheless, van Diemen and colleagues described a key limitation of using single gRNAs to target HCMV genes: the emergence of viral escape mutants [29]. Using single gRNAs inhibited HCMV replication and spread for up to 11 days after infection, but further assessments revealed that after 21 days, some initially blocked viruses began replicating due to mutations in the genome. Singleplex editing of HCMV essential genes UL57 and UL70 primarily resulted in in-frame mutations of the targeted genes that were more likely to allow for expression of functional proteins. The low complexity of the mutant variants also suggested selection of the escape mutants [29]. Overall, the CRISPR-Cas9 method presents a promising strategy to hinder HCMV replication; however, it requires precise viral genome modification to prevent the development and selection of escape mutants that are capable of evading CRISPR-Cas9 editing [29].

### 4.2. Targeting HCMV UL122/123 Using Multiplex CRISPR-Cas Strategy

Taking a different approach to inhibit HCMV replication, Gergen and colleagues utilized the CRISPR-Cas9 system to target the HCMV UL122/123 gene, which encodes the viral immediate-early (IE) proteins IE1 and IE2 (Table 1) [30]. When HCMV is reactivated from its latent phase, IE1 and IE2 are the first to be expressed and play vital roles in activating the expression of viral early and late gene expressions [3,34]. The subsequent expressions of early and late genes are responsible for the assembly and release of new virus particles. In addition to promoting the activation of viral genes, IE1 and IE2 proteins are also able to block host immune defenses that would have otherwise prevented viral replication [3]. IE1 can prevent cell apoptosis, ND-10 suppression of viral genes, and type 1 interferon signaling. Similarly, IE2 blocks cell apoptosis and the production of inflammatory signals like cytokines and chemokines [35]. The vital functions of IE1 and IE2 proteins make them a promising target for combating HCMV lytic infections [36].

In the study described by Gergen and colleagues [30], two strategies were compared: a singleplex CRISPR strategy with one sgRNA, and a multiplex approach where three sgRNAs were used. In the singleplex strategy, the sgRNA was designed to target a region of the UL122/123 gene. For the multiplex approach, three gRNAs were designed to target three different and conserved regions in the UL122/123 gene, specifically near the start codon and at both the beginning and end of exon 5 to ensure the disruption of splice variants of the IE1 and IE2 transcripts. Human MRC5 and MG cells were delivered with the CRISPR-Cas9 system using lentiviral delivery methods and infected with HCMV. The multiplex strategy resulted in the deletion of a large 3300 base pair segment in the UL122/123 gene, which proved more effective than the singleplex strategy [30]. In the multiplex strategy, the expression of both IE1 and IE2 was disrupted by CRISPR-Cas9 targeting UL122/123, with greater levels of reduction in IE2. This disruption was also associated with reduced production of new viral particles and lowered expression of glycoprotein B, a late viral antigen whose expression is dependent on IE1 and IE2 proteins [30]. Overall, the impaired progression of the viral replication cycle and the strong suppression in the expression of IE1, IE2, and late viral proteins highlighted the ability of multiplex CRISPR-Cas9 approaches to target specific genes and effectively inhibit HCMV replication.

### 4.3. Inhibition of HCMV Lytic Replication and Reactivation by the CRISPR-Cas System

Similarly, Xiao and colleagues reported the use of the CRISPR-Cas9 method to directly target the DNA encoding HCMV IE proteins IE1 (p72) and IE2 (p86) (Table 1) [31]. In their study, they utilized a lentiviral vector (i.e., lentiCRISPRv2) that could simultaneously express Cas9 and sgRNAs. A human codon-optimized version of the *S. pyogenes* Cas9 and specifically designed sgRNAs were employed to target conserved regions of the HCMV IE1 and IE2 genes [31]. Cas9 and the sgRNAs were introduced into HCMV-infected human foreskin fibroblasts (HFFs) via lentiviral delivery. The results showed that the CRISPR-Cas9 system caused significant decreases in HCMV UL122 mRNA and IE1 (p72) protein expression [31]. Additionally, these results also revealed significant decreases in the expression of UL54 (an early gene that encodes viral DNA polymerase) and UL83 (a late gene that codes for pp65, an HCMV virion protein) [3,37], alongside a reduction in viral DNA level. These results implied that the inhibition of IE1 and IE2 expressions mediated by CRISPR-Cas9 targeting reduced the expression of viral early and late genes and decreased viral DNA levels and replication [31].

To investigate if the CRISPR-Cas system also inhibits HCMV reactivation, Cas9 and the sgRNAs were introduced into cells from the human THP-1 monocytic cell line, a semi-HCMV latent infection model [3]. These THP-1 cells carrying the CRISPR-Cas system were then infected with HCMV and then treated with TPA to induce HCMV reactivation and lytic replication. Similarly, decreases in the expression of UL122, IE1, UL54, and UL83 as well as the inhibition of viral DNA production were found in the THP-1 cells expressing sgRNAs, implying that viral reactivation and lytic replication was blocked [31]. These results further suggest IE1 and IE2 genes are effective targets for anti-HCMV therapies and underscore the potential of the CRISPR-Cas method as a precise and effective antiviral strategy to limit HCMV infection.

### 4.4. CRISPR-Cas-Mediated Viral Gene Drive

Walter and Verdin explored another approach for limiting HCMV infection: the development of a CRISPR-Cas9-based gene drive in HCMV [32]. Using gene drive techniques, a specific gene or genetic modification can be propagated through a population at a higher rate than through natural inheritance. In this study, they first tested the viability of generating a gene drive system in HCMV (Table 1) [32]. The gene drive cassette consisted of the Cas9 gene, a guide RNA that targeted the viral UL23 open reading frame (encoding a tegument protein in HCMV that blocks interferon-γ) [38], the mCherry fluorescent reporter, and homology arms-matching regions flanking the UL23 locus to enable homologous recombination. This gene drive cassette was inserted into a plasmid and transfected into human foreskin fibroblasts (HFFs) infected with HCMV. Next, these cells were coinfected with the gene drive virus and HCMV Towne-EGFP virus to induce recombination, where Cas9 would cleave the viral UL23 DNA region and allow the insertion of the gene drive cassette into the Towne virus. Cells infected by the next generation of viruses were observed to carry both red and green fluorescent markers, indicating successful recombination [32]. These findings established the feasibility of generating a gene drive system in HCMV to convert wild-type viruses into new gene drive carriers through homologous recombination.

After demonstrating the proof of concept, Walter and Verdin evaluated the effectiveness of using this strategy to prevent HCMV infection. UL23 inhibits host immune responses by blocking IFN-γ through interacting with Nmi (human N-myc interactor) [38]. Thus, the growth of gene drive viruses with the UL23 knockout mutation was significantly attenuated in the presence of IFN-γ. Most importantly, these attenuated gene drive viruses retained their ability to convert wild-type viruses into gene drive viruses. However, they found that wild-type viruses were able to rescue gene drive viruses by providing functional UL23 protein to block IFN-γ. Despite this, subsequent generations of the virus still demonstrated suppressed viral infection in the presence of IFN-γ [32].

Nonetheless, the ability of some mutants of HCMV to resist conversion into gene drive viruses remains a key concern. Mutations of the Cas9 cleavage site enabled escape mutants to resist conversion, eventually outcompeting the gene drive viruses. To address this, Walter and colleagues explored strategies to minimize the fitness of escape mutants [33]. They designed gRNAs to target evolutionarily conserved regions of viral genes important for HCMV replication and infection so that even if the virus successfully mutated to escape the gene drive, mutations in critical genes would impair viral function and replication. Of these gRNAs, those targeting UL26 and UL35 still allowed for efficient spread of the gene drive into the viral population (Table 1) [33]. Both UL26 and UL35 are important for viral replication because HCMV mutants, with the deletion of the UL26 and UL35 open reading frames, were substantially attenuated in growth in human foreskin fibroblasts in vitro [25]. CRISPR-Cas9-targeting of UL26 resulted in out-of-frame and in-frame mutations that disrupted the overall gene function, resulting in very poor viral replication in human foreskin fibroblasts. Targeting of UL35 mutated the start codon of its long isoform, preventing the virus from making the same version of the protein and thus causing moderately poor viral replication [33]. Over time, as gene drive-resistant viruses emerged, total viral levels also rose. However, the viral titer remained lower than the wild-type levels, suggesting that while these viruses were gene drive-resistant, they carried mutations at the CRISPR target site that resulted in permanent replication defects. Altogether, Walter and colleagues demonstrated that gene drive viruses targeting evolutionarily conserved sequences in HCMV genes important for viral replication can spread effectively into wild-type viruses and limit viral replication [33]. These results highlight the potential of CRISPR-Cas-based gene drives as an effective therapeutic strategy for achieving reduction in HCMV replication.

In summary, these studies revealed that each of the anti-CMV CRISPR-Cas-based approaches has its own unique strengths and weaknesses (Table 2). The approach based on the singleplex CRISPR-Cas method has the strength of simple and easy design and expression, but the weakness of a high probability of developing escape mutations. The approach based on the multiplex CRISPR-Cas method can target multiple genes or make large deletions, leading to better cumulative antiviral efficacy. Moreover, the multiplex approach also has a low probability of developing escape mutations. However, this approach may have a weakness of complicated and difficult design and expression. Finally, the strategy based on gene drive cassettes has the strength of converting wild-type viruses into gene drive virus mutants, but has the weakness of selecting outcompeting resistant viruses. Further investigations into these approaches and strategies will facilitate the development of these CRISPR-Cas-based systems for the study and therapy of HCMV infections and diseases.

## 5. Limitations and Challenges in Using CRISPR-Cas Approaches for Studying and Treating HCMV Infections

CRISPR-Cas technology is a powerful tool for studying gene function and holds great potential as an alternative method for limiting HCMV infections and treating HCMV-related diseases. The CRISPR-Cas system can be delivered via viral and non-viral methods [17,39]. Common viral delivery methods include adeno-associated virus (AAV), lentivirus, and baculovirus; non-viral delivery approaches include electroporation, lipid nanoparticles, polymer-based nanoparticles, and gold nanoparticles (Table 3) [39,40,41,42]. Nonetheless, there are various challenges and limitations associated with CRISPR-Cas-based gene-editing methods.

### 5.1. Off Target Effects

One notable limitation in using the CRISPR-Cas9 method is the possibility of off-target effects (OTE) when unintended regions of the genome are cleaved [43,44]. To address this, ongoing research focuses on engineering Cas9 variants with enhanced specificity. One example of such Cas9 variants includes SpCas9-HF1, which was engineered by mutating four amino acid residues responsible for forming hydrogen bonds with the phosphate backbone of the target DNA [45]. These modifications significantly reduced non-specific interactions, resulting in undetectable off-target activity [44]. Some other examples include evoCas9 and HiFiCas9, which were modified to enhance specificity-altering amino acid residues in the Rec3 domain [16,17,18]. The Rec3 domain is a part of Cas9 and is involved in nucleotide recognition, where it recognizes whether a DNA sequence matches the gRNA [44]. This method desensitizes the Rec3 domain, ensuring that Cas9 will only be triggered if the gRNA matches well with the DNA, thereby increasing specificity and reducing OTEs [40,44]. Additionally, adjusting the sequence of the gRNA has also been demonstrated to decrease OTEs [46].

### 5.2. The Need for a PAM Sequence

PAMs are stretches of DNA located downstream of the target DNA and are essential for Cas9 recognition and cleavage [22]. Without the presence of PAMs, even if the gRNA fully complements the target sequence, Cas9 will not cut the DNA. One of the most used Cas9 variants is spCas9 [22,47]. This enzyme has a short PAM site, 5′NGG3′, making it useful clinically, as it is applicable for targeting diverse genomic sites. However, a major limitation is its large size, which makes it difficult to package into an adeno-associated virus (AAV) vector, a common delivery method used in gene therapy [16,17,18]. Another variant, saCas9, is smaller in size and easier to package into the AAV vector. However, its long PAM sequence of 5′NNGRRT3′ or 5′NNGRR(N)3′ (R = G or A) limits the range of therapeutic target sites it can be applied to [44]. To address this challenge, researchers have engineered a smaller-sized Cas9 (e.g., saCas9) to recognize a shorter PAM site, expanding its range [16,17,18]. Another variant, xCas9, not only demonstrated relatively low OTEs in human cells, but also has the capability of recognizing a broad range of PAMs including NG, GAA, and GAT, which can broaden the therapeutic window, making it a promising candidate in a clinical setting [16,17,18].

### 5.3. Toxicity

When CRISPR-Cas9 introduces DSBs to DNA, it can trigger apoptosis in cells rather than initiating DNA repair, the intended pathway for gene editing [22,47]. In the context of CRISPR-based screening, CRISPR-Cas-induced apoptosis can lead to false positives [43]. Some ways this problem has been addressed is through developing alternative versions of the CRISPR-Cas technology that perform base editing without inducing DSBs. This approach can minimize cellular toxicity while improving the accuracy for CRISPR-Cas screens.

Another effect of DSB-induced toxicity is potential selection for oncogenic cells. Recent studies on human pluripotent stem cells have demonstrated that toxic DNA damage could lead to the activation of p53, a key tumor suppressor protein [44,48]. In response to DNA damage, p53 causes cell cycle arrest before the S phase or G2 phase, providing time for DNA repair [49]. In cases where p53 is mutated, cells with damaged DNA can continue dividing, often leading to the development of tumors and cancers. Moreover, p53 activation can lead to cell apoptosis, meaning that CRISPR-Cas-induced DSB could cause cell death if p53 is functional [44,49]. For this reason, CRISPR-Cas9 targeting may favor the survival of oncogenic cells because mutated or suppressed p53 will not induce apoptosis when presented with DSBs, which poses a serious challenge to its use as a clinical treatment. To mitigate this risk, researchers are investigating alternative CRISPR-Cas systems such as Cas9 nickase (Cas9n) [50], which creates single-stranded breaks instead of DSBs. This approach aims to reduce cellular stress and lowers the likelihood of oncogenic selection [48].

### 5.4. Emergence of Escape Mutants Resistant to the CRISPR-Cas Approaches

It has been known that drug-resistant HCMV strains with escape mutations arise during chemotherapy, especially when the drugs (e.g., ganciclovir) are not in optimal concentrations or the immune responses are weakened or deficient, such as in immunocompromised individuals including organ transplant recipients, cancer patients, and HIV-positive individuals [11,12,13]. Thus, HCMV mutants resistant to CRISPR-Cas approaches are expected to emerge if the CRISPR-Cas based system is not highly effective in blocking viral infection and replication in clinical settings. In their elegant study, van Diemen and colleagues revealed a key limitation of using single gRNAs to target HCMV genes, observing the emergence of viral escape mutants with this singleplex CRISPR-Cas strategy [29]. Their strategy with single gRNAs inhibited HCMV replication and spread for up to 11 days after infection, but further assessment revealed that after 21 days, some initially blocked viruses began replicating due to mutations in the genome.

Interestingly, Gergen and colleagues employed both singleplex and multiplex strategies to target the HCMV UL122/UL123 locus [30]. In their singleplex CRISPR strategy, one sgRNA was used to target a UL122/UL123 region. In contrast, three sgRNAs were used to target different regions of UL122/UL123 in their multiplex approach. The multiplex strategy resulted in the deletion of a large 3300 base pair segment in the UL122/123 gene, which proved more effective than the singleplex strategy [30]. Thus, the multiplex approach may have advantages over the singleplex approach in reducing the likelihood of escape mutations and resistant viral mutants. This is because it is more difficult for HCMV to compensate for genome mutations/deletions at three different positions targeted by the multiplex approach compared to the single indel mutation of the singleplex approach [30]. Moreover, potential large deletional mutations of the HCMV genome by the multiplex approach can result in viral mutants incapable of replicating due to the deletion of the viral-essential genes, thereby making it almost impossible for the virus to develop escape mutants resistant to the multiplex approach [30]. Thus, the multiplex-based strategy is clearly a better choice for preventing the emergence of escape mutations resistant to CRISPR-Cas-based approaches.

One key point in preventing the emergence of HCMV mutants resistant to antiviral treatment is to block viral genome replication and progeny production [11,12,13]. Thus, it is important to improve the efficiency of CRISPR-Cas-based approaches in modifying and cleaving the HCMV genome. The CRISPR-Cas system as a tool has been extensively studied and engineering of different enzymes and gRNAs have been carried out to develop better and more active CRISPR-Cas systems for gene-targeting and genome-editing applications [17,18]. Further studies with the engineered and improved CRISPR-Cas systems and better multiplex targeting designs will help inhibit viral replication and block the emergence of resistant viral mutants.

### 5.5. Potential Challenges and Considerations in Treating HCMV Infections with the CRISPR-Cas Approaches in Clinical Settings

Like all human herpesviruses, HCMV has the ability to establish lifelong infections in the host [3]. During latent infection in the bone marrow, HCMV infects hematopoietic progenitor cells and specifically exploits monocytes and other cells of the myeloid lineage to establish latency [9]. To eliminate the latent viral genome, the CRISPR-Cas system needs to be delivered to these cells that are latently infected with HCMV in vivo. Moreover, whether the CRISPR-Cas systems are active in cleaving and modifying the viral latent genome as well as blocking viral reactivation and lytic replication in these clinically relevant cells needs to be determined. Such studies will reveal if the CRISPR-Cas-based approach can eliminate HCMV-latent genomes as well as inhibit viral latent infection, reactivation, and lytic replication in cells known to be latently infected by HCMV.

As one of the most common opportunistic pathogens, HCMV causes significant morbidity and mortality in immunocompromised individuals [3]. For example, this virus inflicts severe and life-threatening complications in organ transplant recipients [51,52]. Reactivation of HCMV in grafts usually leads to rejection. Many of the adverse effects associated with HCMV infection in transplantation could be reduced or eliminated if HCMV could be removed or have its reactivation blocked [51,52]. To prevent HCMV-associated organ transplantation rejection, CRISPR-Cas systems could be delivered to the organs and tissues and be used to cleave and modify the latent HCMV genome, block viral reactivation, and inhibit lytic replication. Future investigations into these topics will provide insights into the development of the CRISPR-Cas-based genome-editing approach for the treatment and prevention of HCMV infections and associated diseases.

## 6. Conclusions

In this review, we have highlighted many advantages of the CRISPR-Cas-based genome-editing technology, including its simplicity, precision, and versatility. Originally discovered as a natural defense mechanism in bacteria and archaea, the CRISPR-Cas gene-editing system has since evolved into a transformative tool in the scientific world. With several successful cases demonstrating its clinical potential, we aim to understand how the CRISPR-Cas technology can be leveraged to study and treat HCMV infections. We have discussed how it has not only been used to identify essential genes or study HCMV gene function, such as through the CRISPR-Cas-based screens, but also to suppress HCMV infection through targeting essential viral genes.

It is worth noting, however, that like many other gene-targeting tools, the CRISPR-Cas technology comes with limitations in its use for studying and treating HCMV infections and diseases, such as off-target effects, dependence on PAM sites, DNA damage-induced toxicity, emergence of escape mutations, and challenges in eliminating viral latent infection as well as blocking viral reactivation and lytic infection in clinically relevant cells and tissues, such as those for transplantations. Despite these challenges, emerging strategies, such as base editors, gRNA modifications, and alternative Cas variants, offer potential solutions to enhance safety and specificity. Overall, while further research and experimentation is needed for its advancement, the CRISPR-Cas technology shows great promise not only in expanding our understanding of HCMV biology, but also as a novel and effective therapeutic strategy for combating HCMV infection.

## Figures and Tables

**Table 2:** The strengths and weaknesses of different CRISPR-Cas gene-editing strategies for HCMV infection.

	Strengths	Weaknesses
Singleplex CRISPR-Cas	Simple and easy design and expression	Higher probability of escape mutations
Multiplex CRISPR-Cas	Can target multiple genes or make large deletions Lower probability of escape mutations	Complicated and difficult design and expression
Gene drive cassettes	Edits self-propagate to wild-type viruses	Positively selects for outcompeting resistant viruses

**Table 3:** Viral and non-viral delivery tools for the CRISPR-Cas system.

	Delivery System	Advantages	Limitations
Viral delivery tools	Adeno-associated virus (AAV)	Safe and high delivery efficiency, broad serotype specificity	Limited packaging size
	Lentivirus	Large loading capacity, high delivery efficiency, low immunogenicity	High cost, difficult to construct
	Baculovirus	Large loading capacity	Difficult for direct genome manipulation
Non-viral delivery tools	Electroporation	Suitable for any type of CRISPR-Cas9 system	Induces high cell death, limited integration of plasmid DNA into target cells
	Lipid-based nanoparticle	Commercially available, safe	Low delivery efficiency
	Polymer-based nanoparticles	Low immunogenicity, easy to modify	Limited application
	Gold nanoparticle	Stable and biocompatible, can be easily customized	High concentration could be toxic in vivo
